# Paclitaxel-Loaded Magnetic Nanoparticles Based on Biotinylated *N*-Palmitoyl Chitosan: Synthesis, Characterization and Preliminary In Vitro Studies

**DOI:** 10.3390/molecules26113467

**Published:** 2021-06-07

**Authors:** Vlad Constantin Ursachi, Gianina Dodi, Alina Gabriela Rusu, Cosmin Teodor Mihai, Liliana Verestiuc, Vera Balan

**Affiliations:** 1 Faculty of Medical Bioengineering, Grigore T. Popa University of Medicine and Pharmacy of Iasi, 700115 Iasi, Romania; vlad.ursachi@umfiasi.ro (V.C.U.); liliana.verestiuc@bioinginerie.ro (L.V.); 2 Advanced Centre for Research-Development in Experimental Medicine, Grigore T. Popa University of Medicine and Pharmacy of Iasi, 700115 Iasi, Romania; gianina.dodi@umfiasi.ro (G.D.); cosmin-teodor.mihai@umfiasi.ro (C.T.M.); 3 Natural Polymers, Bioactive and Biocompatible Materials Laboratory, Petru Poni Institute of Macromolecular Chemistry, 700487 Iasi, Romania; rusu.alina@icmpp.ro

**Keywords:** magnetic nanoparticles, chitosan, biotin, paclitaxel, drug delivery, viability assay

## Abstract

A considerable interest in cancer research is represented by the development of magnetic nanoparticles based on biofunctionalized polymers for controlled-release systems of hydrophobic chemotherapeutic drugs targeted only to the tumor sites, without affecting normal cells. The objective of the paper is to present the synthesis and in vitro evaluation of the nanocomposites that include a magnetic core able to direct the systems to the target, a polymeric surface shell that provides stabilization and multi-functionality, a chemotherapeutic agent, Paclitaxel (PTX), and a biotin tumor recognition layer. To our best knowledge, there are no studies concerning development of magnetic nanoparticles obtained by partial oxidation, based on biotinylated *N*-palmitoyl chitosan loaded with PTX. The structure, external morphology, size distribution, colloidal and magnetic properties analyses confirmed the formation of well-defined crystalline magnetite conjugates, with broad distribution, relatively high saturation magnetization and irregular shape. Even if the ability of the nanoparticles to release the drug in 72 h was demonstrated, further complex in vitro and in vivo studies will be performed in order to validate the magnetic nanoparticles as PTX delivery system.

## 1. Introduction

A considerable interest in cancer research is represented by development of magnetic nanoparticles based on biofunctionalized polymers for controlled release systems of hydrophobic chemotherapeutic drugs (i.e., Paclitaxel) that can be targeted only to the tumour sites, without affecting normal cells [1,2]. These ‘‘smart’’ nanocomposites include: (i) a magnetic inorganic material that is able to direct the systems to the target by magnetic-field-mediated guidance and/or for molecular imaging; (ii) a polymeric surface shell that provides stabilization in physiological environment and multi-functionality; (iii) a chemotherapeutic agent (adsorbed/hosted within internal cavities of the particles) [3]; and (iv) a recognition layer of the tumour cells. The most used magnetic materials are represented by iron oxides (magnetite, or its oxidized form—maghemite) due to their excellent magnetic properties, such as high magnetic saturation and superparamagnetic behaviour, biocompatibility and biodegradability [4,5].

Among polymers, chitosan stands out as a successful polysaccharide endowed with advantageous features, widely involved in the design of magnetic drug-delivery nanosystems with promising results [6,7]. However, these nanosystems need to be functionalized in order to selectively target the cancer cells. To enhance chitosan-targeting ability, various chemical modifications of polysaccharide and its derivatives with active compounds, such as folic acid [8], transferrin [9] and biotin have been proposed [10,11]. A recent review paper highlighted the importance of chitosan-functionalized nanocarriers on enhancing the bioavailability of taxanes (Paclitaxel and Docetaxel) at targeted tumour site [12].

Biotin is an essential vitamin for cells growth and proliferation, being involved in cellular carbohydrate, lipid and amino acid metabolism [13,14]. It is known that biotin-receptors are overexpressed in different tumour cells, including breast, ovarian and lung cancer [15,16]. Consequently, the biotin functionalization approach has been explored as an attractive strategy for cancer targeted-drug and gene-delivery systems [17,18,19]. In this regard, in a previous study, our group synthesized biotinylated *N*-palmitoyl chitosan and evaluated its suitability to formulate polymeric self-assembled nanoparticles for breast cancer therapy [20,21].

Paclitaxel (PTX) is a standard chemotherapeutic drug for breast, pancreatic and non-small-cell lung carcinoma, but its pharmaceutical formulation (Taxol^®^) exerts serious side effects [22]. Therefore, various nanoformulations of PTX are currently under research, with some of them being clinically successful [23].

The objective of this paper is to present the synthesis, characterization and preliminary drug delivery evaluation of PTX loaded magnetic nanoparticles based on biotinylated *N*-palmitoyl chitosan. To our best knowledge, there are no studies concerning development of magnetic nanoparticles obtained by partial oxidation method, based on biotinylated *N*-palmitoyl chitosan loaded with PTX.

## 2. Results and Discussions

### 2.1. Paclitaxel-Loaded Magnetic Biotinylated N-Palmitoyl Chitosan Nanoparticles Preparation

**Figure 1:**
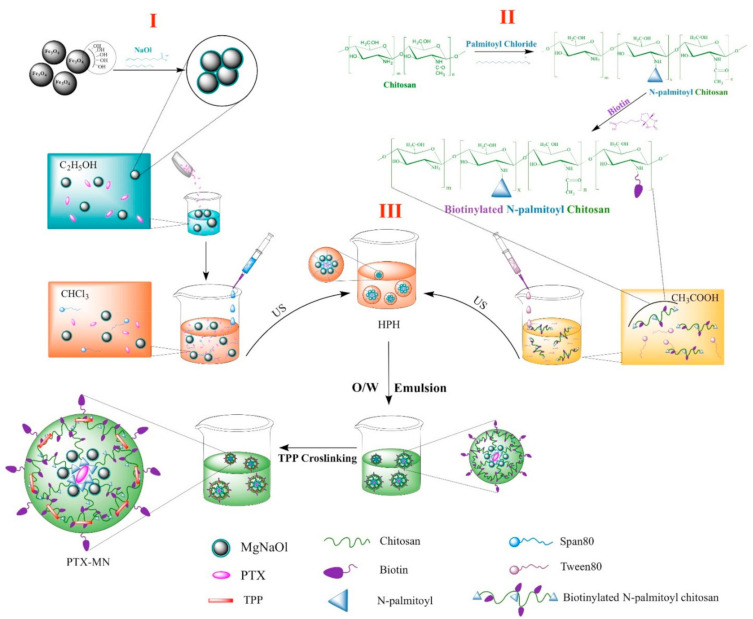
Schematic diagram of the composite magnetic nanoparticles synthesis.

PTX-loaded composite magnetic nanoparticles have been prepared by oil-in-water emulsion procedure. Figure 1 illustrates the schematic representation of the synthesis method divided into three steps, which are succinctly described below.

First step (I): hydrophobic iron oxide nanoparticles preparation by partial oxidation method, followed by embedment within sodium oleate matrix; the procedure yielded well-crystallized, uniformly sized bare magnetite nanoparticles of about 14 nm (according to TEM pictures), higher phase purity (as from X-ray diffraction patterns) and saturation magnetization of 88.3 emu/g. The FTIR analysis confirmed that sodium oleate is partially chemically bonded to the Fe_3_O_4_ nanoparticle, offering hydrophobicity to the bare surface (graphics shown in a previously published paper of the authors) [24].

Second step (II): biotinylated *N*-palmitoyl chitosan formulation via carbodiimide chemistry; the presence of the new methylene groups from palmitoyl chain and new amide bonds formed in the biotinylated polymer, when compared with native chitosan, were confirmed by a combination of spectroscopic investigations, namely FTIR and ^1^H-NMR (graphics shown in a previously published paper of the authors) [20].

Third step (III): a simple oil-in-water emulsion comprising the oil phase containing the hydrophobic magnetite nanoparticles and hydrophobic drug (Paclitaxel), and the aqueous phase including biotinylated functionalized polymer (BPCs). Finally, the mixture was cross-linked with TPP solution and the solidification of nanoparticles was achieved by solvent evaporation under ambient conditions. Further, we discuss in detail the specific features of the PTX-MN biofunctionalized nanoparticles.

### 2.2. Nanoparticles Features

#### 2.2.1. Size, Surface Charge and Morphology

**Figure 2:**
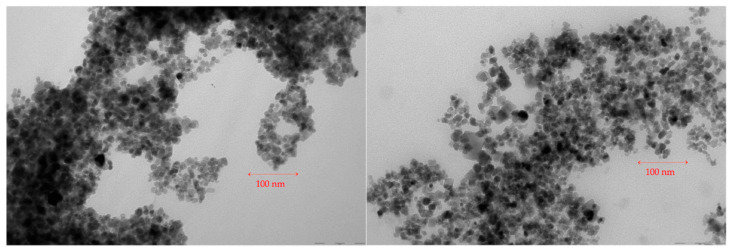
TEM micrographs of PTX-MN nanoparticles.

The magnetic nanoparticles morphology of PTX-MN was investigated by TEM and is shown in Figure 2. As previously mentioned above, bare magnetite nanoparticles produced by oxidation method were found to be 14 nm (S.D. = 4 nm) with a nearly spherical shape [24]. The architecture of the encapsulated Fe_3_O_4_ nanoparticles was reorganized after the functionalization with sodium oleate, coverage with biotinylated *N*-palmitoyl chitosan and PTX loading. The two TEM images of PTX-MN (A and B) showed irregular spherical nanoparticles similar in size with non-coated ones, with smooth surface, arranged to form grape-chain-like structures. TEM micrographs of dried PTX-MN confirmed that magnetite (black spots) was effectively entrapped into biofunctionalized polymer structure.

Dynamic light-scattering data showed an average hydrodynamic size of 205.3 ± 0.64 nm with a polydispersity index of 0.288 and a negative Zeta potential value of −18.7 ± 1.24 mV for PTX-MN; meanwhile, the same parameters registered for MN were 215.7 ± 8.92 nm, a polydispersity index of 0.370 and a Zeta potential value of −19.4 ± 0.40 mV, respectively. The variances in size in aqueous solution compared with dried state of drug-free and loaded composite nanoparticles may be due to a combination of Lifschitz–van der Waals and magnetic forces that aggregate the magnetite nanoparticles into considerably large nanoparticle clusters [25].

#### 2.2.2. Composition and Thermal Behavior

**Figure 3:**
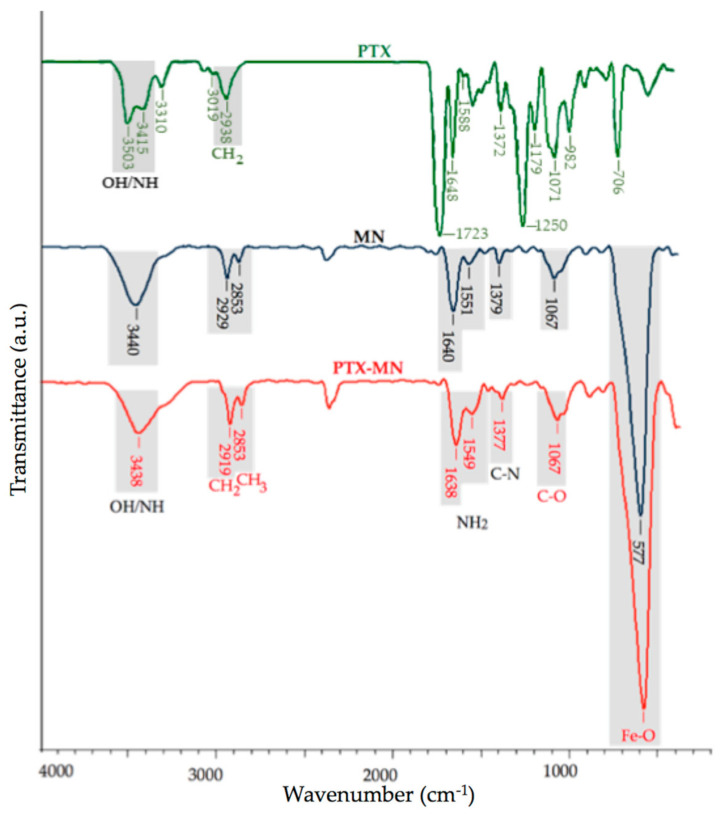
FTIR spectra of PTX, drug-free MN nanoparticles and PTX-MN nanoparticles.

The obtained PTX-MN nanoparticles were characterized by infrared spectroscopy (Figure 3). The main infrared peaks of the PTX were registered at 3415–3310 cm^−1^ (N-H stretching vibrations), 2938 cm^−1^ (methylene stretching vibration), 1720 cm^−1^ (C=O stretching vibration of the ester groups), 1648 cm^−1^ (amide I), 1372 cm^−1^ (the C-N vibrations), 1071 cm^−1^ (C-O stretch), 982 cm^−1^ and 706 cm^−1^ (aromatic bonds) [26,27]. The FTIR spectrum of MN showed the characteristic bands for biotinylated *N*-palmitoyl chitosan at 3440 cm^−1^ (O-H, N-H stretch, imidazole ring of biotin), 2919 cm^−1^ and 2853 cm^−1^ (methylene and methyl groups stretching vibrations from palmitoyl chain), 1640 cm^−1^ and 1549 cm^−1^ (amide I), 1379 cm^−1^ (the C-N vibrations), 1067 cm^−1^ (C-O stretch) and for magnetite at 577 cm^−1^ (specific region for the Fe-O group).

In the FTIR spectrum of PTX-MN the same bands as drug-free nanoparticles with no significant changes can be observed, except the signals arising from N-H stretching, N-H bending and CH stretching. These could be explained by the fact that PTX exhibits common bands with the polymer in the specific regions of O-H, N–H, C=O and CH_2_ groups, respectively [26]. In the same time, it is presumed that PTX is embedded between the magnetic core and the polymer layer.

**Figure 4:**
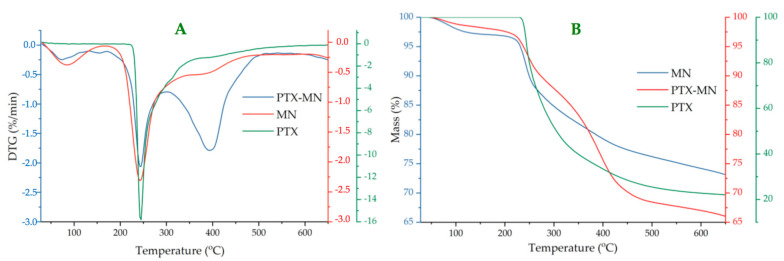
DTG (**A**) and TG (**B**) curves of PTX, PTX-MN nanoparticles and drug-free MN nanoparticles.

Thermogravimetric analyses (Figure 4) of composite magnetic nanoparticles have been performed in order to determine the magnetite content of nanoparticles, based on residue percentage (magnetite is a thermo resistant material). In the same time, by studying the thermo behavior of PTX-MN, relevant data concerning their structure could be obtained, if compared with its components (biotinylated polymer, magnetite and drug) thermo degradation steps. The residue percentage from TG data (around 72% for MN and 66% for PTX-MN, respectively) was correlated with magnetite content.

DTG curve of drug-free nanoparticles (MNs) exhibited three weight loss steps with temperature (Figure 4A): first degradation step with a little weight loss of 3% (at 85 °C) can be attributed to the water evaporation, physically adsorbed or absorbed in the inner polymeric network, whereas the second step with a weight loss of 15% (at 243 °C) and the third step with a weight loss of 10% (at 381 °C) can be assigned to the degradation of polysaccharide units, palmitoyl chains and biotin [20]. Thermodegradation of PTX took placed at 243.6 °C (with a weight loss of 78.22%), in agreement with other data from the literature [22,26].

Thermodegradation of PTX-MN also occurred in three steps: the first one (72 °C) is due to water evaporation; meanwhile, the other two, registered at 244 °C (with a weight loss of 12%) and at 397 °C (with a weight loss of 21%), respectively, could be correlated with the degradation of the biotinylated polymer [20] and the drug highly encapsulated in the magnetic structure [22,26]. Thermogravimetric data confirmed the entrapment of both drug and magnetite in the structure of the nanoparticles.

#### 2.2.3. X-ray Diffraction Analysis

**Figure 5:**
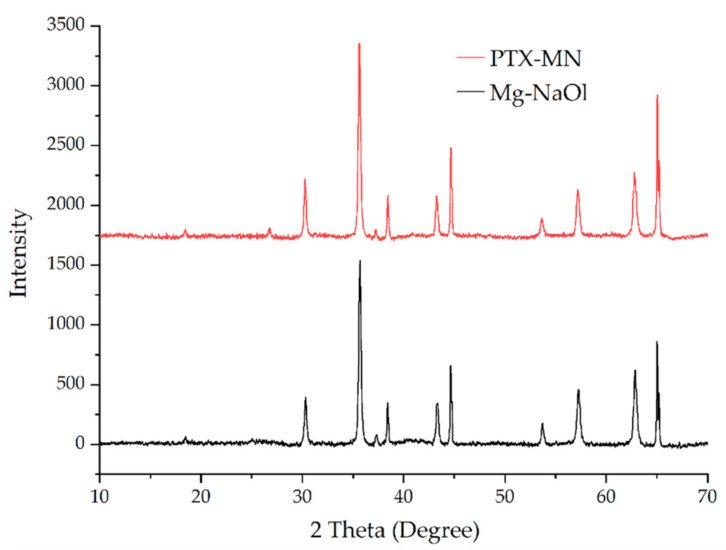
XRD diffractograms of Mg-NaOl and PTX-MN.

The crystalline structure of the Mg-NaOl and PTX-MN was analyzed by powder X-ray diffraction (XRD) and comparative patterns are shown in Figure 5. All the evidenced peaks were indexed by using Joint Committee on Powder Diffraction Standards (JCPDS) database, card no. 19-0629 (pure Fe_3_O_4_). Both X-ray diffraction patterns exhibited sharp peaks with strong diffraction intensity at 2θ values of 18.6°, 30.4°, 35.7°, 43.2°, 53.7°, 57.3° and 62.5° that can be indexed to the (111), (220), (311), (222), (400), (422), (511) and (440) planes, respectively, characteristic to the face centered cubic structure of magnetite (Fe_3_O_4_). After the coating/functionalization/drug incorporation steps, the position and relative intensity of diffraction peaks suggest the preservation of the magnetite nanoparticles structure within the matrix.

#### 2.2.4. Magnetic Properties

Superparamagnetic features and high magnetic saturation are important parameters for magnetic based nanoparticles used in biomedical applications.

**Figure 6:**
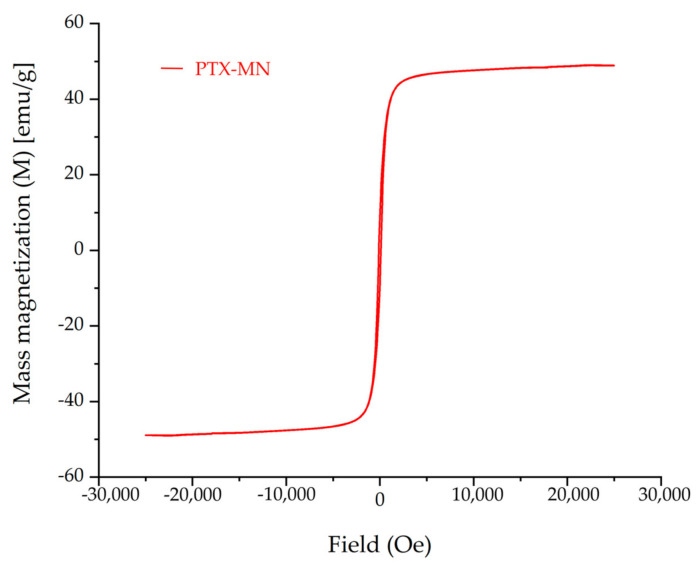
Magnetization curve of PTX-MN.

The magnetization curve of PTX-MN, as depicted in Figure 6, revealed that the nanoparticles exhibited superparamagnetic behavior (no hysteresis was recorded; residual magnetization and the coercive force were zero) and good magnetic saturation of 48.9 emu/g, in agreement with the high content of magnetite determined from TG data.

#### 2.2.5. CytoViva Results

**Figure 7:**
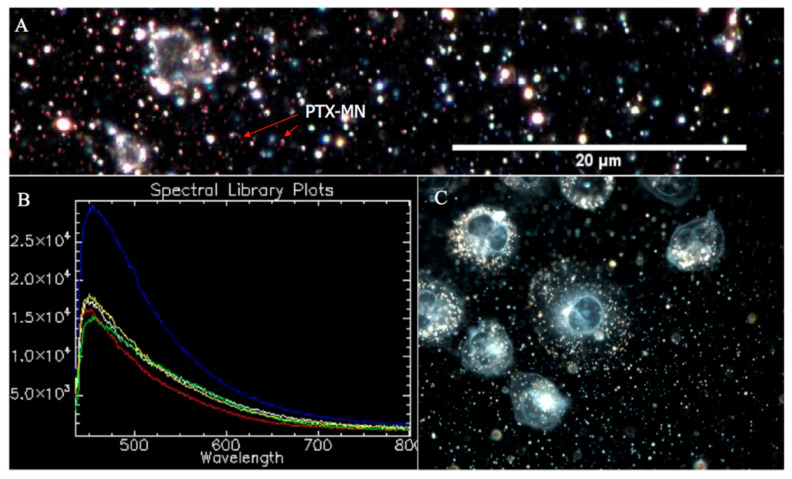
CytoViva hyperspectral microscopy data: (**A**) PTX-MN spectrum, filtered against drug-free nanoparticles spectrum (inset); (**B**) PTX-MN mapping; and (**C**) MDA-MB-231 cells in the presence of PTX-MN.

CytoViva hyperspectral microscopy data, as presented in Figure 7, confirmed the PTX entrapment inside the composite magnetic nanoparticles by spectral mapping of drug unique pixels (Figure 7B). Succinctly, according to the technique procedure, the image was processed by selecting few image pixels, compared with a spectral library of PTX, filtered against drug-free nanoparticles spectrum (Figure 7A, inset) and, consequently, PTX was easily identified based on its characteristic spectrum (blue curve from Figure 7A). Moreover, the CytoViva hyperspectral enhanced dark field images of MDA-MB-231 cells (Figure 7C recorded by using 60× magnification) indicated the presence of PTX-MN (scattered bright white light) inside the cells.

### 2.3. In Vitro Drug Release and Biodegradation Studies

**Figure 8:**
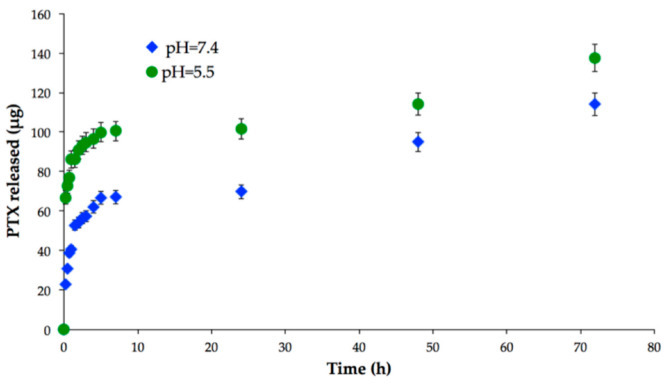
In vitro drug release of PTX-MN.

To assess the potential of PTX-MN as drug-delivery nanoplatforms, preliminary *in vitro* release studies were performed in PBS at different pH values that mimic the conditions of normal cells (neutral pH) and cancer cells (acidic medium), respectively (Figure 8). It is important to mention that drug loading of the composite nanoparticles with PTX could not be determined, since the loading takes place in the synthesis of the material and the complexity of the reaction medium does not allow this analysis of the supernatant. This issue will be resolved by dialysis method, followed by centrifugation, in order to determine the drug encapsulation efficiency. The release profile of PTX evidenced a pH-dependent behavior: in acidic medium, a higher quantity of drug was released, due to the more intense degradation of polymeric matrix in acidic pH (see also Figure 8). In the same time, the vehicles exhibited a biphasic drug release pattern: a fast release in the first 6 h, followed by a second phase corresponding to a sustained drug release up to 72 h. The initial burst effect could be attributed to the diffusion of PTX absorbed onto the surface of nanoparticles or weakly interacted with the hydrophobic cores of the biotin-N-palmitoyl chitosan magnetic nanoparticles, whereas the following gradual release could be endorsed to the diffusion of strongly entrapped drug into the inner hydrophobic domains [28].

**Figure 9:**
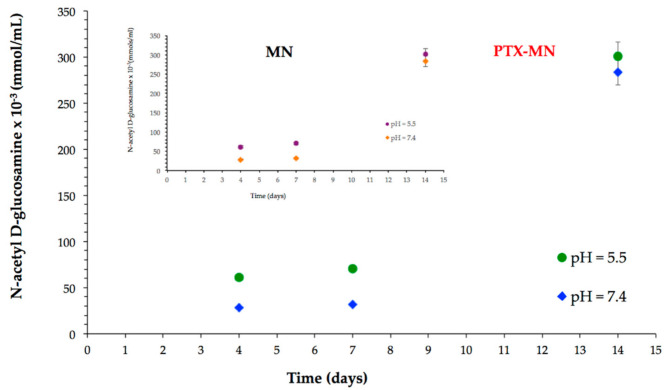
Biodegradation data of PTX-MN and MN.

Biodegradability is an important desideratum of nanomaterials designed for biological applications. It is known that chitosan and its derivatives are exposed to both chemical and enzymatic degradation in the human biological fluids and tissues. The biodegradability studies have been performed in neutral (pH = 7.4) and acidic environment (pH = 5.5) in the presence of lysozyme-the enzyme that will hydrolyze the glycosidic linkages between sugar residues of polysaccharide [28,29], and, consequently, chitosan oligomers and N-acetyl-D-glucosamine units will be released in the medium [30]. N-acetyl-D-glucosamine units formed were quantified by ferricyanide method [31]. The results, as presented in Figure 9, confirmed a good susceptibility of PTX-MN to biodegradation, expressed more intense in acidic medium, due to a higher relaxation of the polymer backbone. The biodegradation behavior of drug-free MN magnetic nanoparticles (inset Figure 9) is similar with that of PTX-MN.

### 2.4. In Vitro Cell Investigations

**Figure 10:**
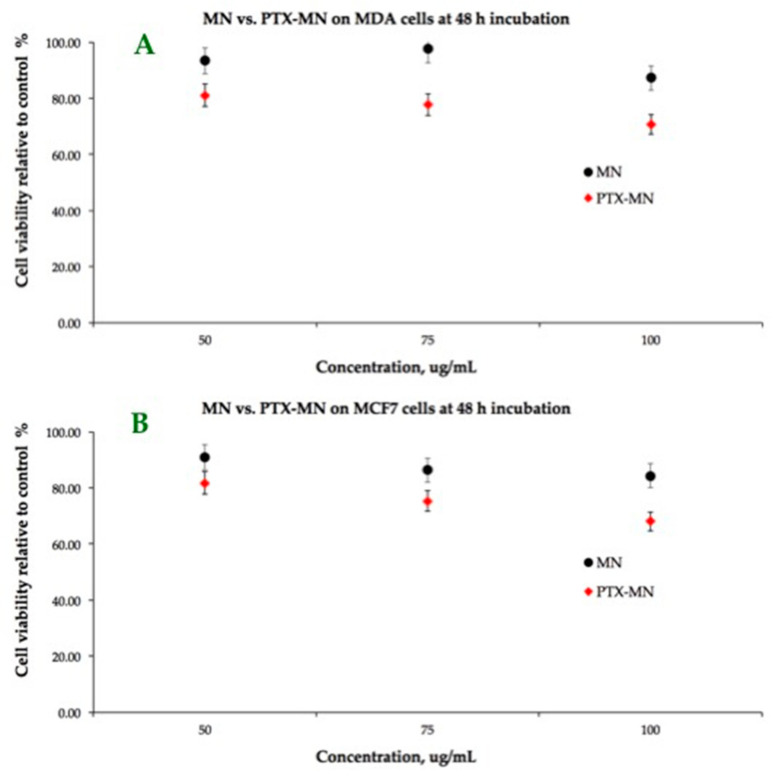
Cellular viability-MTT assay on MCF-7 and MDA-MB-231 cell lines of MN (**A**) and PTX-MN nanoparticles (**B**).

The results of dose-response cytotoxicity tests carried out on MCF-7 and MDA-MB-231 cell lines by using MTT after 48 h incubation with drug-free (MN) and PTX-MN nanoparticles are shown in Figure 10. As expected, the cell-viability data at 48 h after incubation of the magnetic nanoparticles prepared in the absence of PTX are not cytotoxic for the tested concentrations (from 50 to 100 μg/mL), showing more than 80% of metabolically active cells. The cell viability was found to decrease by more than 15% on both cell types when using the drug-loaded magnetic nanoparticles based on biotinylated *N*-palmitoyl chitosan, due to the PTX release into the medium. If we correlate the *in vitro* drug-release data with cell viability assay, the amount of released PTX in both acidic and neutral media at 48 h was reduced (in the first step only the surface absorbed drug was released). It is important to mention that these preliminary studies had the limitation of poor dispersion into the medium, since the release investigations were done in PBS solution, favorable for the nanoparticle design. Further studies will be done in order to assess the release into the different compatible mediums for both cells and nanoparticles, in order to improve the drug-release profile in a simulated cellular substrate.

## 3. Materials and Methods

### 3.1. Materials

Biotinylated *N*-palmitoyl chitosan (BPCs) and hydrophobic magnetite nanoparticles (Mg-NaOl) used for the composite materials synthesis were prepared according to previous reports of our group, briefly detailed in the next section [21,24]. Sodium tripolyphosphate (TPP), Paclitaxel isolated from *Taxus brevifolia*, sorbitan monooleate (Span 80), polyoxyethylene sorbitan monooleate (Tween 80), DMEM-low glucose (Dulbecco’s Modified Eagle Medium), FBS (fetal bovine serum, suitable for cell culture, sterile-filtered), PBS (phosphate buffered saline solution, sterile-filtered), MTT (3-(4,5-dimethyl-2-thiazolyl)-2,5-diphenyl-2H-tetrazolium bromide), Trypan Blue (0.4%), chloroform, glacial acidic acid and ethanol were purchased from Sigma-Aldrich. Lysozyme (from a chicken egg) was obtained from Fluka. The MCF-7 cell line specific to human Caucasian breast adenocarcinoma and MDA-MB-231 human breast adenocarcinoma cell line were acquired from the European Collection of Cell Cultures (ECACC).

Analytical grade chemicals were used as received, without further purification. All solutions were prepared with double-distilled water.

### 3.2. Synthesis of Paclitaxel-Loaded Magnetic Nanoparticles Based on Biotinylated N-Palmitoyl Chitosan

As already mentioned above, the first two steps of the composite materials synthesis (see Figure 1) were prepared according to our previously published papers.

Briefly, in the first step, hydrophobic iron oxide nanoparticles (Mg-NaOl) were prepared in a two-phase procedure: partial oxidation of iron (II) chloride tetrahydrate (3.78 mmoles) in alkaline solution (0.5 M aqueous ammonia solution), using nitrate ion as a mild oxidizing agent (10%), followed by bare nanoparticle functionalization (1%) in aqueous suspension, using sodium oleate (1%) under specific conditions of temperature and nitrogen atmosphere [24].

Secondly, biotinylated *N*-palmitoyl chitosan (BPCs) has been obtained through the reaction of *N*-palmitoyl chitosan with biotin, via carbodiimide chemistry in two steps:

*N*-palmitoyl chitosan with a degree of substitution of 12.35% (as determined by trinitrobenzene sulphonic acid assay—TNBS) has been obtained through the reaction of free amine groups of low molecular weight chitosan with the acyl groups of palmitoyl chloride through nucleophilic acyl substitution; then, biotin dissolved in dimethyl sulfoxide–water mixture solution and activated with EDAC and NHS was reacted with *N*-palmitoyl chitosan (1% in 0.1 M acetic acid) to obtain biotinylated *N*-palmitoyl chitosan derivative [20].

Thirdly, PTX-loaded magnetic nanoparticles based on biotinylated *N*-palmitoyl chitosan have been obtained by oil-in-water emulsion, as follows: first, hydrophobic phase I containing 1 mL hydrophobic magnetite (1%) solution, 0.1 mL Span 80 and 5 mg PTX in 4 mL chloroform and hydrophilic phase II comprising 0.1 mL Tween 80 in 10 mL BPCs (0.5%) were mixed by using ultrasound irradiation (Bandelin ultrasonic homogenizer, 200 W nominal output, continuous pulse (cycle 2) and 100% amplitude of ultrasonic agitation for 1 min). Then, the two phases were emulsified using high-pressure homogenization (HPH) with IKA T25 Ultraturax (3 × 5 min, 15,000 rpm). After adding 2 mL of aqueous TPP (0.5%) solution, the mixture was mechanical stirred vigorously for 24 h to evaporate the organic solvent. Paclitaxel loaded magnetic nanoparticles based on biotinylated *N*-palmitoyl chitosan (further referred as PTX-MN) were collected by centrifugation at the 10,000 rpm and purified with deionized water. Drug-free magnetic nanoparticles (MNs) were prepared in the same conditions, in the absence of drug, and used as reference in characterization techniques.

### 3.3. Characterization of Composite Magnetic Nanoparticles

The morphology of magnetic based nanoparticles was studied by transmission electron microscopy on a dry sample (FEI Tecnai F20 field emission, high-resolution transmission electron microscope operating at an accelerating voltage of 200 kV and equipped with Eagle 4k CCD camera). Zeta potential and hydrodynamic mean diameter of the obtained nanoparticles were determined by dynamic light-scattering measurements in water, at 25 °C, using a Malvern ZetasizerNanoS (Malvern Instruments, Malvern, UK). A Vertex 70 (Bruker) FTIR spectrophotometer was used to record FTIR spectra in all samples. The recording of the results took place at a resolution of 4 cm^−1^ in the range of 500–4000 cm^−1^. Thermogravimetric analysis (TGA) and differential thermal analysis (DTA) of the samples were evaluated in dynamic conditions using STA 449 F1 Jupiter apparatus (Netzsch, Selb, Germany), in nitrogen atmosphere, at a 10 °C min^−1^ heating rate, on the temperature interval situated between 30 and 650 °C. X-ray diffraction (XRD) patterns (*2*θ range 0°–70°) were analyzed on a Shimadzu XRD-6000 diffractometer, using Ni-filtered CuK_α_ radiation (λ = 1.5418 Å) at a scanning speed of 2 °C/min. Particles’ magnetization was measured by using a vibrating sample magnetometer (Lakeshore VSM 7400 System), at 25 °C and ±20,000 G applied magnetic field. In order to confirm the drug entrapment into the nanostructures, high-resolution enhanced dark-field image and hyperspectral signal image were obtained via CytoViva Enhanced Darkfield Hyperspectral Microscope (CytoViva, Auburn, AL, USA). The images were processed to identify the spectral signature of PTX by selecting a few image pixels that were compared with a spectral library of PTX. The MDA-MB-231 cells were exposed to a 20 μL droplet of magnetic nanoparticles (50 μg/mL) and imaged by using a CytoViva Enhanced Darkfield Hyperspectral Microscope (Auburn, AL, USA).

### 3.4. In Vitro Drug Release Studies

In vitro release profiles of PTX from the magnetic nanoparticles were studied in two phosphate buffered saline (PBS) mediums (at pH values 7.4 and 5.5). A weighed amount of freeze-dried PTX-MN was redispersed in PBS and transferred into a dialysis bag (molecular weight cutoff 12,400 Da) and placed in preheated PBS (total volume: 25 mL). The release study was performed in an incubator shaker, at 37 °C, for 72 h. At selected time intervals (15, 30, 60, 90, 120, 150, 180, 240, 300 and 360 min; 24, 48 and 72 h), the solution outside of the dialysis bag was removed (*n* = 3) for analysis by UV–Vis Spectroscopy (UV-1700 PharmaSpec, Shimadzu, Kyoto, Japan) at 227 nm and replaced with fresh buffer solution.

### 3.5. Biodegradability Studies

The susceptibility of magnetic nanoparticles to enzymatic degradation was assessed in two simulated media (PBS at pH 7.4 and 5.5), using the ferricyanide method. A weighed amount of composite magnetic nanoparticles was dispersed in 2 mL PBS containing 3 mg of lysozyme and transferred into a dialysis bag (molecular weight cutoff 12,400 Da), placed in a preheated PBS (total volume of 25 mL) and incubated at 37 °C for 6 days. At selected time intervals, aliquots (of 1 mL) from the solution outside of the dialysis bag were withdrawn, mixed with 4 mL potassium ferricyanide solution (0.5 M), quenched in boiling water (100 °C; 15 min) and made up to 10 mL with buffer. Following rapid cooling to 25 °C (within 5 min; ice bath), the optical absorbance was recorded at 420 nm. After 3 days, the medium was completely removed and replaced with fresh medium. An N-acetyl-D-glucosamine calibration curve was used to assess the amount of chitosan-reducing ends driven into solution at each time point.

### 3.6. Cell Culture Assays

For cytotoxicity tests, MCF-7 and MDA-MB-231 cell lines were used and cultured according to the producer’s protocol. Briefly, the cells were grown in DMEM medium supplemented with 10% fetal bovine serum, 100 μg/mL streptomycin and 100 IU/mL penicillin. For this study, 5 × 10^3^ cells/well were cultivated in 96-well tissue culture plates at a temperature of 37 °C, in a humidified atmosphere containing 5% CO_2_. The magnetic composites nanoparticles (both types) were suspended in the above-specified medium at different concentrations (100, 75 and 50 μg/mL) and applied to the culture wells in 200 µL volume. The cells were incubated for 48 h in the same conditions and standard MTT assay was performed on the samples, using Tecan Sunrise Plate Reader, at 570 nm. The results are expressed as a mean ± standard deviation (SD) (*n* = 3).

## 4. Conclusions

This study proposed for the first time, a method to obtain PTX loaded magnetic nanoparticles obtained by partial oxidation based on biotinylated *N*-palmitoyl chitosan. The physical–chemical features of the magnetic nanoparticles, in terms of size, surface charge, morphology and composition, were evaluated. FTIR spectroscopy combined with thermal analyses and hyperspectral microscopy data confirmed the structure of the composite magnetic nanoparticles: polymer, magnetite and PTX. In the same time, the obtained drug-loaded polymeric magnetic nanoparticles showed high magnetic saturation and superparamagnetic behavior. Preliminary in vitro studies indicated the ability of the nanoparticles to release the PTX up to 72 h, with a more pronounced drug release within acidic media and susceptibility to biodegradation in simulated biological fluids. Further complex in vitro and in vivo studies need to be performed in order to accomplish the goal of obtained magnetic nanoparticles as a PTX delivery system.

## Sample Availability

Samples of the compounds are not available from the authors.
